# Brownian reservoir computing realized using geometrically confined skyrmion dynamics

**DOI:** 10.1038/s41467-022-34309-2

**Published:** 2022-11-15

**Authors:** Klaus Raab, Maarten A. Brems, Grischa Beneke, Takaaki Dohi, Jan Rothörl, Fabian Kammerbauer, Johan H. Mentink, Mathias Kläui

**Affiliations:** 1 Institut für Physik, Johannes Gutenberg-Universität Mainz, Staudingerweg 7, 55128 Mainz, Germany; 2 Radboud University, Institute for Molecules and Materials, Heyendaalseweg 135, 6525 AJ Nijmegen, The Netherlands; 3 Graduate School of Excellence Materials Science in Mainz, Staudingerweg 9, 55128 Mainz, Germany

**Keywords:** Magnetic properties and materials, Ferromagnetism, Computational nanotechnology

## Abstract

Reservoir computing (RC) has been considered as one of the key computational principles beyond von-Neumann computing. Magnetic skyrmions, topological particle-like spin textures in magnetic films are particularly promising for implementing RC, since they respond strongly nonlinearly to external stimuli and feature inherent multiscale dynamics. However, despite several theoretical proposals that exist for skyrmion reservoir computing, experimental realizations have been elusive until now. Here, we propose and experimentally demonstrate a conceptually new approach to skyrmion RC that leverages the thermally activated diffusive motion of skyrmions. By confining the electrically gated and thermal skyrmion motion, we find that already a single skyrmion in a confined geometry suffices to realize nonlinearly separable functions, which we demonstrate for the XOR gate along with all other Boolean logic gate operations. Besides this universality, the reservoir computing concept ensures low training costs and ultra-low power operation with current densities orders of magnitude smaller than those used in existing spintronic reservoir computing demonstrations. Our proposed concept is robust against device imperfections and can be readily extended by linking multiple confined geometries and/or by including more skyrmions in the reservoir, suggesting high potential for scalable and low-energy reservoir computing.

## Abstract (web summary)

Magnetic skyrmions, due to their strongly nonlinearity and multiscale dynamics, are promising for implementing reservoir computing. Here, the authors experimentally demonstrate skyrmion-based spatially multiplexed reservoir computing able to perform Boolean Logic operations, using thermal and current driven dynamics of spin structures.

## Introduction

Skyrmions are magnetic whirls with topologically enhanced stability, which can behave like two-dimensional quasi-particles^1,2^. Skyrmions have been found in thin metal films^3–7^ and bulk materials^8^, and the efficient displacement due to spin-transfer-torques^9^ and spin-orbit-torques (SOT)^10–14^ by low current densities bears tremendous potential for non-conventional computing^15–20^ and novel types of memory devices^21,22^.

While deterministic skyrmion dynamics due to current-induced torques have previously been exploited^12^, recently, it has been shown that skyrmions exhibit stochastic dynamics induced by thermal diffusion^17^. This dynamics is intrinsically nonlinear as well and, moreover can be easily influenced by geometrical confinement^23,24^, where the equilibrium configurations of skyrmion arrangements can be controlled by geometry. In particular, by exploiting commensurability effects, the ordering of the equilibrium arrangements can be tailored^24^. Furthermore, the current-induced torques have so far not been systematically combined with thermally excited diffusion, but potentially this combination can reduce the current densities for current-induced motion dramatically. Exploiting skyrmion motion for non-conventional computing^25^ has been considered highly promising due to the combination of both intrinsic nonlinear dynamics and stochastic dynamics, which are both features of the human brain as well^26^.

One of the key paradigms in brain-inspired computing is reservoir computing (RC)^27^, which leverages the nonlinear dynamics of a medium to map a complex problem to a much simpler linear problem. Since the reservoir itself does not need to be trained, training costs reduce to that of solving a linear problem, yielding fast and low-energy learning^28^. Although several theoretical proposals exist for implementing RC with magnetic skyrmions, they heavily rely on the existence of local pinning sites^15,29^, and the nonlinear dynamics feature small displacements, making read-out challenging experimentally. Moreover, for extended magnetic skyrmions textures, the reproducible operation requires an external reset mechanism. On top of that, the skyrmion dynamics relying on the deterministic motion induced by high current densities^17^ limits the potential to improve energy efficiency as compared to existing spintronic RC^30^.

We overcome these challenges by designing and experimentally realizing a conceptually new approach: a Brownian skyrmionic device as an RC component, which exploits the intrinsic properties of thermally active skyrmions in geometrical confinement combined with ultra-low power current-induced dynamics. An effective potential well created by the confinement allows for a natural reset mechanism, which does not rely on pinning effects, but is instead enabled by the thermal fluctuation of the skyrmions in combination with the geometrical confinement itself. To demonstrate the functionality of this RC device, we exemplify reliable and reproducible Boolean logic operations, including the non-linearly separable operation.

## Results

### Functionality of the device

**Fig. 1:**
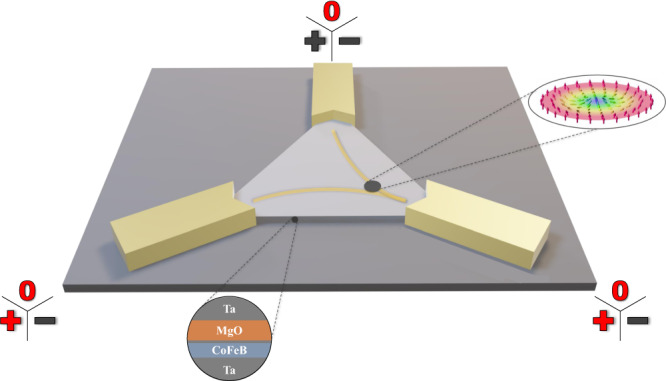
Three-dimensional schematic of the device. The stack structure is shown in the lower circle. Attached are the chromium/gold contacts, on which positive, negative, or null potential can be applied. The input values used for the present Boolean logic demonstration are highlighted in red. The dark gray spot in the triangle represents a skyrmion as imaged in our magneto-optical Kerr effect (MOKE) microscopy recordings. Here, it is pushed into the lower right corner (yellow lines represent schematically the current flow). The inset on the right depicts the schematic spin structure of a Néel-type skyrmion. Note: Inset adapted from the original of Karin Everschor-Sitte and Matthias Sitte, which is licensed under Creative Commons Attribution-Share Alike 3.0 Unported (CC BY-SA 3.0).

Figure 1 schematically shows the system under consideration, in which a confinement geometry of an equilateral triangle harbors a single skyrmion (sample and fabrication details are given in the methods section). This simple and highly symmetric geometry allows for a variety of different states (skyrmion positions) depending on the voltages applied to the contacts at the three corners of the triangular confinement. The device is channeled with narrow wires at the tips for better connectivity to the gold pads. When voltage potentials are applied to the contacts, the skyrmion position depends on the interplay between current-induced motion due to SOTs^10–14^, skyrmion-edge repulsion^23,24^, and thermal diffusive dynamics^17^. The state of the system is imaged by magneto-optical Kerr-effect microscopy (imaging setup description in the methods section). To evaluate the performance of the system as part of an RC device, we track the skyrmion positions and mimic read-out via magnetic tunnel junctions (MTJs) as described below. In principle, many input combinations are possible, however, to resemble the Boolean functions below, we restrict ourselves to grounding one contact and employing ground or positive voltage values as inputs 0 or 1, respectively, at the other contacts (Fig. 1).

**Fig. 2:**
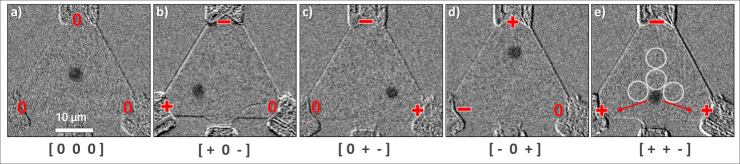
Skyrmion displacement. Subtracted Kerr-microscopy images of an equilateral triangular device with an edge length of 36 µm (tip to tip), each with a single skyrmion (dark gray) in **a** the ground state without and **b**–**e** with applied electrical potentials. In brackets below are the input patterns of the respective state. **e** includes exemplarily the four regions used to mimic MTJs in our analysis. Red arrows show the most probable directions in which the skyrmion will move due to thermal excitation. The chromium/gold contacts overlap the thin film in the corners and on the bottom edge (the latter is unused for the results shown).

The skyrmions are nucleated as described in a previous work^17^ by applying an in-plane magnetic field pulse on top of a static out-of-plane field at the spin reorientation transition. One of the main advantages of this device is the automatic initialization: by, ideally, nucleating a single skyrmion in the device, the system immediately relaxes to the required ground state as the skyrmion resides primarily in the central region of the confinement due to the skyrmion-edge repulsion^23,24^ as shown in Fig. 2a. Thereby and in particular without the necessity of further input or adjustment of the local pinning effect^15,29^, the ground state, in which the system resets itself when no potential is applied, is achieved. Thus, the interplay of thermal dynamics and edge repulsion acts as an auto-initialization and reset mechanism simultaneously.

### Current-induced skyrmion dynamics

As seen in Fig. 2a, the skyrmion without any applied voltage rests in the middle region of the triangle. For example, by applying a positive potential at one corner, a negative at an opposite corner, and ground (null potential) to the remaining one, we observe the current-induced motion of the skyrmion towards the corner with the applied positive potential. We observe current-induced motion starting at 5 × 10^7^ A m^−2^ in a direction opposite to the direction of the current flow, which is consistent with previous work but at a current density that is about four orders of magnitude smaller^17^. This drastically reduced threshold current density allows for ultra-low power operation. As the current density is increased, the effects of the torques become more and more dominating until increasing the current density further eventually results in pushing the skyrmion into one of the corners, where the current density is highest (see supplementary material), and the skyrmion annihilates. Here we choose a current density in the range of 10^8^ A m^−2^, which is sufficient for reliable and quick operation while not resulting in any skyrmion annihilation events.

Since the current density increases monotonically towards the tip of the triangle element, we calculate the current density value of the device at the half-width of the triangle at the corner from which the current originates. While we have a geometrically symmetric system, the resistance between the pairs of contacts/corners slightly differs due to fabrication irregularities. Hence, the same voltage potential leads to different current density values, which, in addition to possible pinning sites, influences the skyrmion occurrence. A deviation from the ideal equilateral triangle does not impede operation, since the thermally activated diffusive dynamics allow the skyrmions to explore the full state space. Such irregularities are accounted for by default in the training of the linear read-out (see detailed discussion in the supplementary material). Hence, we demonstrate that our RC method automatically compensates for sample/setup imperfections. A detailed discussion on how thermal effects can leverage device imperfections and thereby drastically increase the robustness and reliability of operation can be found in the supplementary material.

### Evaluation and linear regression

The general approach is to manipulate the skyrmion dynamics via the applied electric potentials and measure the probability for the skyrmion to be present within different regions of the sample. For now, the read-out is performed optically by imaging and tracking the skyrmions. For a scaled-down device, this could, for instance, be technically done using average tunnel magnetoresistance (TMR), as the TMR depends on the presence of a skyrmion in the relevant region^31^. To mimic read-out via MTJs^31^, we employ four circular read-out regions with a radius of 2,2 µm within the confinement arranged center-symmetrically as indicated in Fig. 2e. The effects of the skyrmion size on the read-out are discussed in detail in the supplementary material. The images in Fig. 2b–e show only exemplarily the displacement of a single skyrmion from the ground state in Fig. 2a. Note, that we have studied multiple devices that all show qualitatively the same behavior. The results shown below stem from a device with a 40 µm edge length.

The local skyrmion occurrence probabilities within the four circular regions are processed externally via linear read-out, i.e., the output is given by the weighted sum of the probabilities plus an offset. Thereby, the same device can perform a multitude of different operations depending on the weights.

**Fig. 3:**
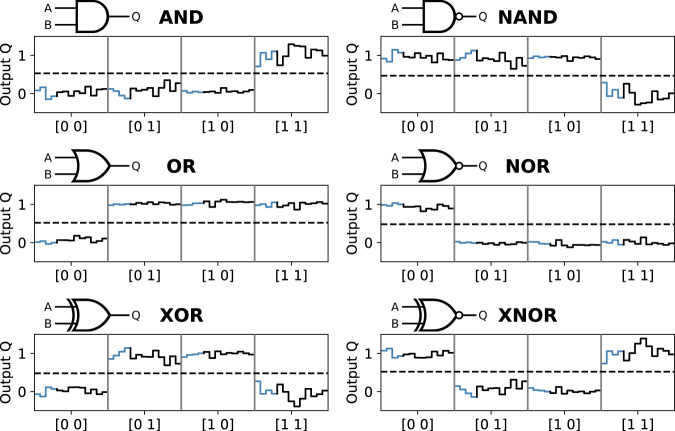
Logic operations. Outputs of the linear read-out optimized for different Boolean operations. For each input combination ([0 0], [0 1], [1 0], [1 1]) the output Q of the linear read-out is shown for 13 sets of local skyrmion occurrence probabilities. The light blue and black parts of the curves indicate the sets used for training and testing, respectively. The dashed horizontal line indicates a possible threshold for perceptron read-out.

The skyrmion occurrence is measured using Kerr-microscopy for 13,000 frames at 16 frames per second. To obtain a series of local occurrence probabilities for the four regions, we average the skyrmion occurrence over 62.5 s time intervals. For each input combination, we use the first 4 of the resulting 13 sets of local occurrences probabilities to optimize (train) the weights for linear read-out using the Scikit-learn software package^32^. The Output *Q* is then given by

1 $$Q={W}_{{{{{{\rm{left}}}}}}}{P}_{{{{{{\rm{left}}}}}}}+{W}_{{{{{{\rm{right}}}}}}}{P}_{{{{{{\rm{right}}}}}}}+{W}_{{{{{{\rm{top}}}}}}}{P}_{{{{{{\rm{top}}}}}}}+{W}_{{{{{{\rm{middle}}}}}}}{P}_{{{{{{\rm{middle}}}}}}}+{W}_{{{{{{\rm{intercept}}}}}}},$$

where ${W}_{{{{{{\rm{region}}}}}}}$ is the weight of the probability ${P}_{{{{{{\rm{region}}}}}}}$ for the skyrmion to be in the specific circular region and ${W}_{{{{{{\rm{intercept}}}}}}}$ is an offset. The probability ${P}_{{{{{{\rm{region}}}}}}}$ itself depends on the input patterns of the device ([0 0], [0 1], [1 0], and [1 1]) (see Fig. 3).

Figure 3 shows the output of the linear read-out, trained for the Boolean operations AND, NAND, OR, NOR, XOR, and XNOR. They are performed using the left and right bottom contacts as inputs, 0 and 1, respectively, while constantly keeping the top contact at 0. The training set is indicated as the light blue part of the curves, whereas the test set is black. For all Boolean operations, we observe values close to the expected output and a good separation even on the test set. The corresponding optimized weights can be found in the supplementary material (Supplementary Table 1). The dashed lines show possible thresholds for perceptron read-out, i.e., values above and below the threshold are assigned to 1 and 0, respectively. In particular, we have demonstrated NAND and NOR functionality, each representing a functionally complete set of logical connectives, and the non-separable XOR functionality. The latter demonstrates that this system with just one confined skyrmion is already sufficiently complex to perform nonlinearly separable tasks, which is impossible, for instance, using a conventional single-layer perceptron read-out alone.

The signal-to-noise ratio (${{{{{{\mathrm{SNR}}}}}}}$) of the read-out is defined as

2 $${{{{{\bf{{{SNR}}}}}}}}\,:=\,\frac{\left\langle {{{{{\boldsymbol{T}}}}}}\right\rangle -\left\langle {{{{{\boldsymbol{F}}}}}}\right\rangle }{{{{{{{\boldsymbol{\sigma }}}}}}}_{{{{{{\boldsymbol{T}}}}}}}+{{{{{{\boldsymbol{\sigma }}}}}}}_{{{{{{\boldsymbol{F}}}}}}}},$$

where T and F are the sub-sets of linear read-out outputs ${{{{{\rm{Q}}}}}}$, for which the corresponding Boolean operation applied to the device input patterns give True or False, respectively. Angled brackets indicate the mean value and ${{{\upsigma }}}_{{{{{{\rm{T}}}}}}}$ (${{{\upsigma }}}_{{{{{{\rm{F}}}}}}}$) is the standard deviation of the T (F) subset. Averaged over the six different Boolean operations, the data in Fig. 3 exhibits ${{{{{\rm{SNR}}}}}}\; > \;5$.

The ${{{{{\rm{SNR}}}}}}$ decreases as the time interval used to obtain the local skyrmion occurrence probabilities is decreased. For example, if the time interval is divided by two while the ratio between the number of sets used for training and testing is kept constant, the ${{{{{\rm{SNR}}}}}}$ decreases to ${{{{{\rm{SNR}}}}}}\approx 4$. The corresponding signal of the logic operations can be found in supplementary Fig. 6. Note here that the timescale of skyrmion diffusion is exponentially dependent on the sample temperature^17^, which is chosen to create diffusion compatible with the sampling rate of the Kerr microscope. A read-out via magnetic tunnel junctions would allow overcoming this limitation. Additionally, the diffusion is faster for smaller devices, as the characteristic timescale of directed diffusive motion scales with the square of the corresponding length scale^33^. Thus, a nanometer scale device would allow for very fast measurements, as discussed in the Supplementary Material.

By using additionally the top contact of the device as a variable input, we can also realize three-input composite logic operations as demonstrated in supplementary Fig. 7. The average SNR of the three-input operations with SNR >3 is still slightly lower than for the two-input operations but still demonstrates reliable operation.

## Discussion

While the presented Brownian RC already suffices to realize nonlinearly separable operations for a single confined skyrmion, we emphasize that the concept can easily be generalized by exploiting more input combinations and by increasing the number of skyrmions in the geometry. With higher numbers of skyrmions, the system exhibits more complex dynamics like (in-)commensurable ground states^24^ and thus enhances the accessible states for the RC. Moreover, the complexity can be further enhanced by linking multiple confined geometries, which could provide an ultra-low-energy alternative to neuromorphic computing based on arrays of nanoscale spintronic oscillators^34–36^. Another advantage is the possibility of the combination of the all-magnetic linear read-out with in-memory computing, for example, magnetic random-access memory^37^. Furthermore, the scalability of this concept to nanoscale dimensions reduces the displacement distances, which would give rise to latency in the nanosecond regime^17^.

Finally, we want to acknowledge that after completion of our work, we became aware of a related work, which also uses skyrmions for non-conventional computing^38^.

This conceptually new reservoir computing idea based on Brownian dynamics of magnetic skyrmions in confined geometries bypasses challenges for existing theoretical proposals for skyrmion RC. Moreover, leveraging the stochastic motion of the skyrmion allows operation at current densities several orders of magnitude smaller than existing spintronic reservoir computation concepts. The present demonstration is based on a single confined skyrmion, which is already sufficient to realize non-separable operations and the universal set of Boolean functions. Generalizing this concept to multiple confined skyrmions and scaling to nanoscale dimensions allow for a highly promising path to ultra-low energy neuromorphic computing.

## Methods

### Sample parameters

The thin film layer stack used was sputtered by a Singulus Rotaris magnetron sputtering system, consisting of Ta(5)/Co_20_Fe_60_B_20_(0.95)/Ta(0.09)/MgO(2)/Ta(5) with the thickness of the layers in nanometers in parentheses^17^. Subscripted numbers are the relative atomic concentration of the respective element in percentage. The sample is specifically tailored for low pinning and skyrmions above room temperature, which exhibit thermal diffusion. Even though this Cobalt-Iron-Boron-based thin film inherits a very flat energy landscape^17^ and thus low pinning, which also enables thermal diffusion of skyrmions, pinning can influence the position of the skyrmion in relation to the corners.

The structures were patterned by electron beam lithography (EBL) using a Raith Electron Beam Pioneer system and then etched by Argon ion etching using an IonSys Model 500 ion beam etching system. 15 µm long wires in 120° relation to each other at the corners were used in the layout of the triangle to ensure a better electrical connection with the gold pads. The width of the wires is between 2 and 5 µm, the skyrmions used in this work do not enter the wire and stay in the triangle due to the skyrmion-edge repulsion. Different device sizes ranging from 22 to 40 µm edge length were tested, but for the presented results, a device size of 40 µm was used. For the electrical contacts, a lift-off technique was used after EBL was done for the layout of the pads. The contact pads consist of 5 nm of chromium and 60 nm of gold on top and have a base size of 250 µm² × 250 µm². Contacting is established using an aluminum wire which is bonded from the pads to the sample’s homemade holder.

### Measurement setup

The sample itself is placed on a QC-17-1.0-2.5MS Peltier element to achieve the necessary temperature stability at around 330 K at ambient air, measured by a Pt100 resistive heat sensor, to realize the skyrmion phase and the size of skyrmions appropriate for the operation. The achievable temperature range was 285–360 K with temperature stability of 0.3 K. The size of the skyrmions and their thermal diffusion directly depend on the temperature^17^. Albeit necessary for the skyrmions in our stack, the increased temperature also leads to increased diffusion, which thus can lead to a necessity of higher current densities to keep a skyrmion in one corner. If the thermal energy is too high, the skyrmion can jump/move towards the middle or other pinning sites in the device, which can be outside of the measured circles or, in the extreme case, annihilate.

We observe the magnetic structures with a commercial Evico GmbH magneto-optical Kerr effect (MOKE) microscope with a CCD camera connected to a PC. The magnetic out-of-plane field is supplied by a self-built electromagnetic coil, while the in-plane magnetic field pulse is supplied by a rotatable electromagnetic coil from the microscopes’ manufacturer. Images and videos are taken using the polar magneto-optical Kerr effect, recorded by a Hamamatsu Digital CCD Camera (C8484-03G02) with a CCD spatial resolution of 1344 × 1024 pixels. The videos are recorded with a frame rate of 16 frames per second at an exposure time of 62.5 ms. To achieve this frame rate, we use a 2 × 2 binning (four physical pixels are averaged to one virtual pixel in the image), resulting in a resolution of 672 × 512 pixels with a field of view of 125 × 95 µm. Differential images between skyrmion hosting states and saturation states were used to enhance the contrast. This leads to black-and-white subtraction errors at the edges of the device in the images (owing to incomplete overlapping), which causes an operation error when the sample/structure moves under the microscope due to thermal drift or mechanical strain. We used a stable thermal equilibrated state and increased mechanical stiffness to reduce the drift. Additionally, a script for repositioning the device in the pictures was used to further reduce the thermal and mechanical drift which occurs during recording, thus ensuring more correct tracking.

### Skyrmion imaging and induced motion

Skyrmions are nucleated by setting an out-of-plane (OOP) field in the μT regime and applying an in-plane pulse field of 35 mT, by switching off the field after it saturated the sample for a second. The amount of skyrmions nucleated in the structure depends on the set OOP field, since the radius of the skyrmions can be influenced by the OOP field and the number of skyrmions the geometrical structure can harbor. By increasing the OOP field, the size of the skyrmions is reduced until skyrmions start annihilating and only one single skyrmion is left. Decreasing the OOP field again to a fixed value leads to a reproducible skyrmion size. The skyrmion moves around the center of the device due to thermal motion but is mostly restricted to staying in the center due to the edge repulsion of the device. For a scaled-down version of our device, one could achieve skyrmion nucleation due to spin-orbit torques by current flowing through magnetic tunnel junctions^39,40^, which are also suggested for read-out, thus performing a double purpose.

The electric potentials are applied by two independent voltage sources (Keithley 2400 SourceMeter), which are connected to the ground through a custom-made breakout box. The latter prevents the device from breaking due to unintentional current flow or induction of current in the wires/cables due to the magnetic fields. The breakout box allows to safely apply the voltage potential to the sample.

Depending on the structure, the potentials for the current-induced skyrmion motion range from 2 up to 5.5 mV, which corresponds to a few μA of electrical current. Considering the geometry, the current densities used at the half-width of the triangle range from 2 × 10^7^ A m^−2^ to 3 × 10^8^ A m^−2^. Although the geometry is symmetrical, the resistance between pairs of the corners of the triangle is not exactly the same and varies due to possible film inhomogeneities, the connections of the gold pads, and the varying bonding connection quality of the attached wires. This is compensated by applying different weights in the linear regression, which also takes possible pinning sites in the triangle into account. With increasing current density, the temperature increases, in particular at the tips of the triangle element. The increased temperature enhances the motion of the skyrmion and its likelihood of annihilating, if the current density, and thus the temperature, becomes too high.

### Image analysis

For the analysis of the videos, the skyrmion in the device was tracked using the trackpy package for Python^41,42^ and its center position for every frame was compared to the overlaying mask of the four center-symmetric circles on the device. The number of frames, in which the skyrmion resides in a certain circular region are summed up and put into relation to the overall number of frames in the video, resulting in the probability of the skyrmion being in a certain area while a fixed current is applied. In the supplementary material, we employ a more elaborate scheme to calculate the occurrence probabilities taking into account skyrmion size effects; we demonstrate that the consequent changes in the occurrence probabilities have little effect on the operation of the device. Figure 1 in the supplementary is the heatmap of the probability of a position for a single skyrmion in the device. One can see that the likelihood at certain positions is increased, indicating the existence of pinning sites^43^, on which it is energetically favorable for the skyrmion to stay. Thermal activation leads to motion around said sites. This thermal activation can be increased using a higher temperature, until the skyrmion becomes either too small for our experimental setup’s resolution or annihilates.

As an outlook, using the three different input types with three physical inputs, 27 possible combinations would be possible, although some combinations have either no function (e.g., [- - -]) or are redundant due to gauge invariance of the electric potential (e.g., state combination (a) - - 0 and (b) 0 0 +, resulting in the skyrmion to move towards the corner with (a) 0 or (b) +). When more skyrmions are present in confinement, the systems’ responses to the inputs become more complex. This is expected to be most prominent for skyrmion numbers incommensurate with the geometry. Additionally, combining multiple devices would lead to even more states, thus leading to even higher capacity^37^.

## Supplementary information

Supplementary Information

## Data Availability

The data supporting the findings of this work are available from the corresponding authors upon reasonable request.

## Code availability

The computer codes used for data analysis are available upon reasonable request from the corresponding author.

## References

[1] Fert A, Reyren N, Cros V (2017). Magnetic skyrmions: advances in physics and potential applications. Nat. Rev. Mater..

[2] Bogdanov AN, Panagopoulos C (2020). Physical foundations and basic properties of magnetic skyrmions. Nat. Rev. Phys..

[3] Dohi, T., Reeve, R. M. & Kläui, M. Thin film skyrmionics. *Annu. Rev. Condens. Matter Phys*. **13**, 73–95 (2022).

[4] Everschor-Sitte K, Masell J, Reeve RM, Kläui M (2018). Perspective: magnetic skyrmions—Overview of recent progress in an active research field. J. Appl. Phys..

[5] Jiang W (2017). Skyrmions in magnetic multilayers. Phys. Rep..

[6] Finocchio G, Büttner F, Tomasello R, Carpentieri M, Kläui M (2016). Magnetic skyrmions: from fundamental to applications. J. Phys. D Appl. Phys..

[7] Wiesendanger R (2016). Nanoscale magnetic skyrmions in metallic films and multilayers: a new twist for spintronics. Nat. Rev. Mater..

[8] Tokura Y, Kanazawa N (2021). Magnetic skyrmion materials. Chem. Rev..

[9] Jonietz F (2010). Spin transfer torques in MnSi at ultralow current densities. Science.

[10] Jiang W (2015). Blowing magnetic skyrmion bubbles. Science.

[11] Woo S (2016). Observation of room-temperature magnetic skyrmions and their current-driven dynamics in ultrathin metallic ferromagnets. Nat. Mater..

[12] Litzius K (2017). Skyrmion Hall effect revealed by direct time-resolved X-ray microscopy. Nat. Phys..

[13] Dohi T, DuttaGupta S, Fukami S, Ohno H (2019). Formation and current-induced motion of synthetic antiferromagnetic skyrmion bubbles. Nat. Commun..

[14] Litzius K (2020). The role of temperature and drive current in skyrmion dynamics. Nat. Electron..

[15] Prychynenko D (2018). Magnetic skyrmion as a nonlinear resistive element: a potential building block for reservoir computing. Phys. Rev. Appl..

[16] Pinna D (2018). Skyrmion gas manipulation for probabilistic computing. Phys. Rev. Appl..

[17] Zázvorka J (2019). Thermal skyrmion diffusion used in a reshuffler device. Nat. Nanotechnol..

[18] Jibiki Y (2020). Skyrmion Brownian circuit implemented in continuous ferromagnetic thin film. Appl. Phys. Lett..

[19] Song KM (2020). Skyrmion-based artificial synapses for neuromorphic computing. Nat. Electron..

[20] Brems MA, Kläui M, Virnau P (2021). Circuits and excitations to enable Brownian token-based computing with skyrmions. Appl. Phys. Lett..

[21] Fert A, Cros V, Sampaio J (2013). Skyrmions on the track. Nat. Nanotechnol..

[22] Nagaosa N, Tokura Y (2013). Topological properties and dynamics of magnetic skyrmions. Nat. Nanotechnol..

[23] Pepper RA (2018). Skyrmion states in thin confined polygonal nanostructures. J. Appl. Phys..

[24] Song C (2021). Commensurability between element symmetry and the number of skyrmions governing skyrmion diffusion in confined geometries. Adv. Funct. Mater..

[25] Li S (2021). Magnetic skyrmions for unconventional computing. Mater. Horiz..

[26] Grollier J, Querlioz D, Stiles MD (2016). Spintronic nanodevices for bioinspired computing. Proc. IEEE.

[27] Tanaka G (2019). Recent advances in physical reservoir computing: a review. Neural Netw..

[28] Marinella MJ, Agarwal S (2019). Efficient reservoir computing with memristors. Nat. Electron..

[29] Pinna D, Bourianoff G, Everschor-Sitte K (2020). Reservoir computing with random skyrmion textures. Phys. Rev. Appl..

[30] Torrejon J (2017). Neuromorphic computing with nanoscale spintronic oscillators. Nature.

[31] Tomasello R (2017). Electrical detection of single magnetic skyrmion at room temperature. AIP Adv..

[32] Pedregosa F (2011). Scikit-learn: machine learning in Python. J. Mach. Learn. Res..

[33] Einstein A (1905). Über die von der molekularkinetischen Theorie der Wärme geforderte Bewegung von in ruhenden Flüssigkeiten suspendierten Teilchen. Ann. Phys..

[34] Romera M (2018). Vowel recognition with four coupled spin-torque nano-oscillators. Nature.

[35] Zahedinejad M (2022). Memristive control of mutual spin Hall nano-oscillator synchronization for neuromorphic computing. Nat. Mater..

[36] Houshang A (2016). Spin-wave-beam driven synchronization of nanocontact spin-torque oscillators. Nat. Nanotechnol..

[37] Jung S (2022). A crossbar array of magnetoresistive memory devices for in-memory computing. Nature.

[38] Yokouchi T (2022). Pattern recognition with neuromorphic computing using magnetic field–induced dynamics of skyrmions. Sci. Adv..

[39] Yang S (2021). Electrical generation and deletion of magnetic skyrmion‐bubbles via vertical current injection. Adv. Mater..

[40] Büttner F (2017). Field-free deterministic ultrafast creation of magnetic skyrmions by spin–orbit torques. Nat. Nanotechnol..

[41] Allan, D. B., Caswell, T., Keim, N. C., van der Wel, C. M. & Verweij, R. W. Trackpy: fast, flexible particle-tracking toolkit — trackpy 0.5.0 documentation. soft-matter/trackpy: Trackpy v0.5.0 10.5281/zenodo.4682814 (2021).

[42] Crocker JC, Grier DG (1996). Methods of digital video microscopy for colloidal studies. J. Colloid Interface Sci..

[43] Gruber R (2022). Skyrmion pinning energetics in thin film systems. Nat. Commun..

