# Supplementary material for: Brownian reservoir computing realized using geometrically confined skyrmion dynamics
Source: Nat Commun. 2022 Nov 15;13:6982. doi: 10.1038/s41467-022-34309-2 (PMC9666653; doi:10.1038/s41467-022-34309-2)
Supplement: Supplementary file 1 — Supplementary Information [file 41467_2022_34309_MOESM1_ESM.pdf]

Supplementary Information

Energy comparison and computation time estimate

To give a rough estimate of the lower limit of energy consumption of the device, we assume that we can scale it down to the nanometer regime with an edge length of 400 nm and a skyrmion size of 40 nm which is a similar ratio of device size and skyrmion size (note that we estimate the size and that the device could technically be manufactured even smaller). The read-out would be done using nanometer sized MTJs. We calculate the resistivity of our manufactured device by approximating the geometry between two connections as a rectangular wire with the width of the inner radius of the triangle (11.5 μm) and the length of 5/6 of the triangle's edge length (33.3 μm, parallel line to one edge going through middle point). The obtained resistivity is then applied to the hypothetical downsized device, which results in a resistance of $R \approx 1$ kΩ. Using the lower limit of the current density of $J = 5\times10^{7}$ A m^{-2} for distinct motion in the down scaled version led to a current flow of 63.5 nA to move a skyrmion. We want to note that for this current density we included the 5 nm Tantalum capping layer, whose purpose is the protection of the layers below from oxidation. In a device with MTJs and filling material on top, these 5 nm Tantalum are not needed and thus, an even lower current density would be required. To estimate the time for one operation of dislocating the skyrmion and releasing it back to the middle, we use the current-induced motion velocities found in Ref.[1] and diffusion constant values of Ref.[2]. The skyrmion has to move up to ~230 nm (outer radius of the triangle) from the center to one corner. We are considering the current-induced motion velocity of Ref.[1] of υ =100 m s^{-1}. The resulting time for the skyrmion to move towards the corner is $t_\mathrm{c} = 2.3\,\mathrm{ns}$. If the current is turned off, we use the diffusion coefficient $D = 70 \times 10^{-9}\ \mathrm{m^2 s^{-1}}$ at ambient temperature from Ref.[2] to calculate the time for the reset of the device

$$t_\mathrm{d} = r^2/4D, \qquad \text{(S1)}$$

where t_d and r represent the required time for the re-set operation and the distance between the center and the corner of the device, respectively. The calculated time is t_d = 190 ns. Hence, the operation speed is mostly limited by the re-set process. Please note that this calculated time is an upper limit as we did not take into account the edge repulsion at the tip of the triangle, which leads to faster motion towards the center of the device again and thus the reset time can be significantly shorter. The exploration time of the device depends on the time required for the skyrmion to diffuse over an equal portion of the sample as in the micrometer regime. This time is proportional to the surface area of the triangle over the skyrmion diffusion constant. Using the sizes mentioned above, the nanoscale diffusion constant from Ref.[2] and a micrometer skyrmion diffusion constant of $D = 1.5 \times 10^{-10} m^2 s^{-1}$ calculated from a previous work Ref. [3], this leads to a required total exploration time of 13.3 µs. The read-out time of MTJs are in the nanosecond or even picosecond regime[4,5] and thus negligible compared to the time required for skyrmions to diffuse a large enough distance to ensure good sampling and therefore allows for even more than 1000 read-outs during one operation.

The electrical power needed for one operation (MTJs excluded) using Power $P = RI^2$ with the current I = 63.5 nA and resistance of 1 kOhm is P = 4 pW. Considering the calculated times before and one operation of pushing a skyrmion into a corner, and the time of reading the skyrmion positions, the energy per oscillation/operation is $E = P \cdot (t_c + t_{readout}) = \ 54\ a\mathrm{J}$. The time required for one Boolean Logic operation would be below $t_{Boolean\ logic\ operation} = t_c + t_d + t_{\mathrm{exploration}} = 13{,}5\ \mu s$. These time and energy requirements could also potentially be further reduced because fewer frames/signals already lead to a good result, as shown in Supplementary Figure 6. Note that we exclude here the energy consumption of the MTJs and any CMOS overhead so that the numbers have to be considered as only part of the total energy consumption. Compared to the consumption of the down scaled version of the spin-torque

oscillator of Ref.[6] of hundreds of Attojoule per oscillation, the calculated energy value seems promising.

Necessity of thermal diffusion

In a real device, thermal fluctuations can be exploited to leverage imperfections and thereby drastically increase the robustness and reliability of the operations. In our device specifically, thermal skyrmion diffusion leverages pinning effects and resistance asymmetries as described in the following. Consider 3 inputs in a device without Brownian motion (top is always ground): [left, right] = [++], [0+] and [+0]. Inputs [0+] and [+0] push the skyrmion into the right and left corner, respectively. In a perfect device (no pinning, completely symmetric resistances, and therefore current profiles), [++] would consistently push the skyrmion to the downward middle as shown for illustrative purposes in one snapshot in Fig. 2e. However, if the resistance of the device is inhomogeneous/asymmetric due to fabrication, as it is in most devices including ours (see “Discussion and interpretation of the weights for linear read-out” above and supplementary Fig. 4a), even at [++] the skyrmion will be pushed into just one corner, in our case the left one. Then input [++] is indistinguishable from input [+0]. With thermal dynamics, the skyrmion will only predominantly be pushed into the left corner but can still overcome the asymmetry and diffuse to the energetically less favorable, right corner. Thereby, the signal is now again distinguishable from [+0] where the probability to be in the left corner is much higher (See Supplementary Fig. 4a, bottom row and Supplementary Fig. 1.). Thus, thermal dynamics can leverage imperfections and thereby significantly enhance the robustness of the system.

A similar example can be made for skyrmion pinning: considering a device without Brownian motion and where resistance asymmetries are either not present or corrected by adjusting input voltages. Skyrmion pinning has been shown to induce strongly inhomogeneous effective

energy-landscapes for skyrmions[40] (One can also spot such pinning sites in Supplementary Fig. 1.). Thus, the skyrmion would not move to a fixed downward middle position but instead the non-flat energy landscape would act as a symmetry breaking mechanism and the skyrmion would take the path of lowest energy (highest pinning) into one of the corners where the current is higher compared to the downward middle (See Supplementary Fig. 2). Again, Brownian motion can leverage this effect and reintroduce distinguishable configurations for all three inputs ([++], [0+] and [+0]).

Discussion and interpretation of the weights for linear read-out

As the weights are optimized using linear regression on 4 of the 13 sets of local occupation probabilities, a given set of local skyrmion occurrence probabilities in the four regions, which is a consequence of the applied input voltages, is mapped to either 0 or 1 depending on the desired output for the given Boolean operation for each input. Therefore, the values of weights in Table 1 can be understood taking into account the global skyrmion occurrence distribution in Sub. Fig. 1. Consider the OR operation: The output should be 1 for input-voltages [0 mV, 2 mV], [2 mV, 0 mV] and [2 mV, 2 mV] at the [left, right] corners of the triangle and 0 for [0 mV, 0 mV]. In the former cases, occurrence is always high in the left and/or right region and therefore the weight is positive, whereas occurrence in the top region indicates a [0 mV, 0 mV] input which should be mapped to 0 and therefore the weight for the top region is negative. In principle, since the results of the Boolean operations are invariant under an input transformation [A, B] to [B, A] and since the read-out regions are placed symmetrically around the vertical mirror axis of the triangle, the weights for the left and right regions should be identical. However, due to asymmetries in the resistance between the contacts and pinning effects, they are not. This does not hinder the functionality of our device, since the weights automatically adjust for these effects. Moreover, note that the weights (except for the intercept)

for NOT operations are just the negatives of their counterpart, as the not operation only exchanges if the result should be mapped to 0 or 1.

Since the [0 mV, 0 mV] input combination is the only one generating high local occurrence probability in the top region (supplementary Fig. 1), the operations which only need to separate this input from the other ones (i.e., OR and NOR) show reduced noise as seen in Fig. 3.

Impact of skyrmion size effects on the modelled TMR read-out

In the main text, TMR read-out is modelled by measuring the probability for the skyrmion center to be in one of four circular regions. Thereby, the effect of the skyrmion size and partial overlap between the area covered by the skyrmion and the area of the TMR read-out element is not well described. Since the read-out regions and the skyrmion are of approximately equal size, the TMR signal will depend on the overlap of the skyrmion area and the read-out area. To incorporate skyrmion size effects, we adapt the calculation of the P_{region} by weighting the occupation counts for each frame with the relative areal overlap between the read-out region and the skyrmion (here, the skyrmion and the read-out area are assumed to have approximately the same diameter). These weighted counts are divided by the total number of frames to obtain P_{region}. In addition, this implementation takes into account that, due to the proximity of the read-out regions, a single skyrmion in a single frame can contribute weighted occupation counts to multiple read-out regions. In applying this method, the occupation probabilities are altered as shown in supplementary Fig. 4. These changes can be well understood when considering the spatially resolved skyrmion occurrence probability in Supplementary Fig. 1: For example, there evidently is a rather strong pinning site located right inside the boundary of the right region, which explains a reduction of P_{right} when calculated using the new method. However, the pattern of occupation probabilities for each Boolean input is still easily

distinguishable, such that the skyrmion size effect is barely noticeable after optimizing the linear read-out (Supplementary Fig. 5). The signal-to-noise ratio is not significantly changed, which shows the robustness of the studied system. Note that the aforementioned reduction of P_{right} reflect in an increase of W_{right} (see Supplementary Table 2).

3-input operations realized using the same system

By using the top contact of our device as an additional input, we can perform 3-input composite logic operations. Supplementary Fig. 7 depicts several 3-input operations using the same system as for the 2-input Boolean operations. Note that the choice read-out regions are different for the 3-input operations to account for the different induced skyrmion dynamics. All operations in supplementary Fig. 7 show reliable operation with perceptron read-out and a high average SNR >3, which is only slightly lower than the 2-input SNR>5. We have employed here the above discussed overlap-based read-out modelling.

Current density distribution

To understand why and where skyrmions are annihilated as the current is increased, a minimalistic COMSOL Multiphysics®[7] simulation has been performed. Only the 5 nm top tantalum layer is simulated, and the gold/chromium contact is modelled as a chromium pad enclosing the sides (but not the top) of the rectangular extensions at the corners. Experimentally, we observed that with a higher current density, the skyrmion would first get pushed into the corner and then get pulled into the high current spots in the simulation in supplementary Fig. 2. The probability of a skyrmion annihilating was highest.

Supplementary figures and graphs

- Supplementary Table 1: Weights of linear regression
- Supplementary Table 2: Weights of linear regression for overlap-based read-out
- Supplementary Figure 1: Heat maps of skyrmion occurrence
- Supplementary Figure 2: Current density distribution simulation
- Supplementary Figure 3: Multiple skyrmion state
- Supplementary Figure 4: Overlap-weighted regional occupation probabilities
- Supplementary Figure 5: Overlap-weighted logic gates
- Supplementary Figure 6: Logic gates with halved time interval for local skyrmion occurrence probabilities
- Supplementary Figure 7: 3-input operation

Supplementary Table 1| Weights of linear regression

Operator	W_{left}	W_{right}	W_{top}	W_{middle}	$W_{\text{intercept}}$
**AND**	1.124	7.497	0.623	1.022	-0.797
**NAND**	-1.124	-7.497	-0.623	-1.022	1.797
**OR**	0.272	1.09	-1.403	-2.908	0.942
**NOR**	-0.272	-1.09	1.403	2.908	0.058
**XOR**	-0.852	-6.407	-2.027	-3.93	1.739
**XNOR**	0.852	6.407	2.027	3.93	-0.739

Supplementary Table 1| Weights of the read-out for the different positions of the skyrmion for the exemplary Boolean operations.

Supplementary Table 2| Weights of linear regression for overlap-based read-out

Operator	W_{left}	W_{right}	W_{top}	W_{middle}	$W_{\text{intercept}}$
**AND**	2.48	9.735	1.595	2.29	-1.287
**NAND**	-2.48	-9.735	-1.595	-2.29	2.287
**OR**	0.807	1.915	-1.75	-2.4	0.806
**NOR**	-0.807	-1.915	1.75	2.4	0.194
**XOR**	-1.673	-7.82	-3.345	-4.69	2.093
**XNOR**	1.673	7.82	3.345	4.69	-1.093

Supplementary Table 2| Weights of the read-out for the different positions of the skyrmion for the Boolean operations, based on the occupation probabilities calculated using the relative areal overlap of the skyrmion and the read-out regions.

Supplementary Figure 1| Heat maps of skyrmion occurrence

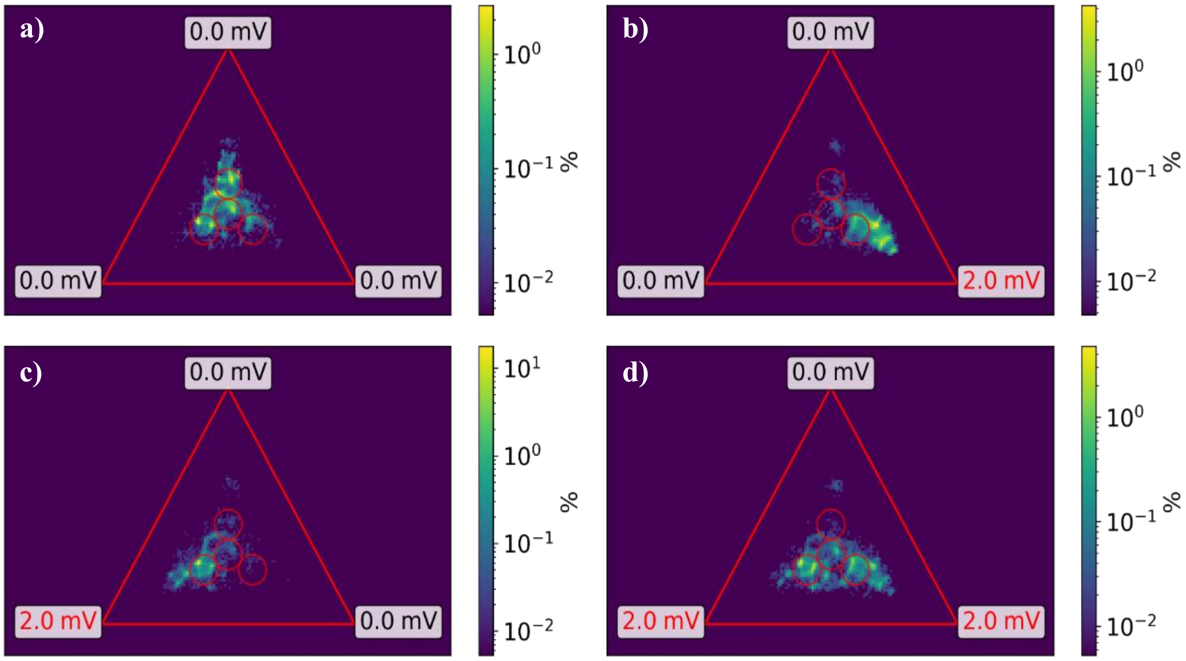

Supplementary Figure 1| Heat maps of the device showing the skyrmion occurrence probability. Yellow represents highest likelihood of a skyrmion position. In the corners the respective, applied potential is shown in either black for the ground potential (0.0 mV) or in red for +2.0 mV potential. A) Free diffusion, b) skyrmion is pushed to the right, c) skyrmion is pushed to the left and d) skyrmion is pushed to the left and right depending on random thermal motion. Red circles are the read-out area used for the linear read-out.

Supplementary Figure 2| Current density distribution simulation

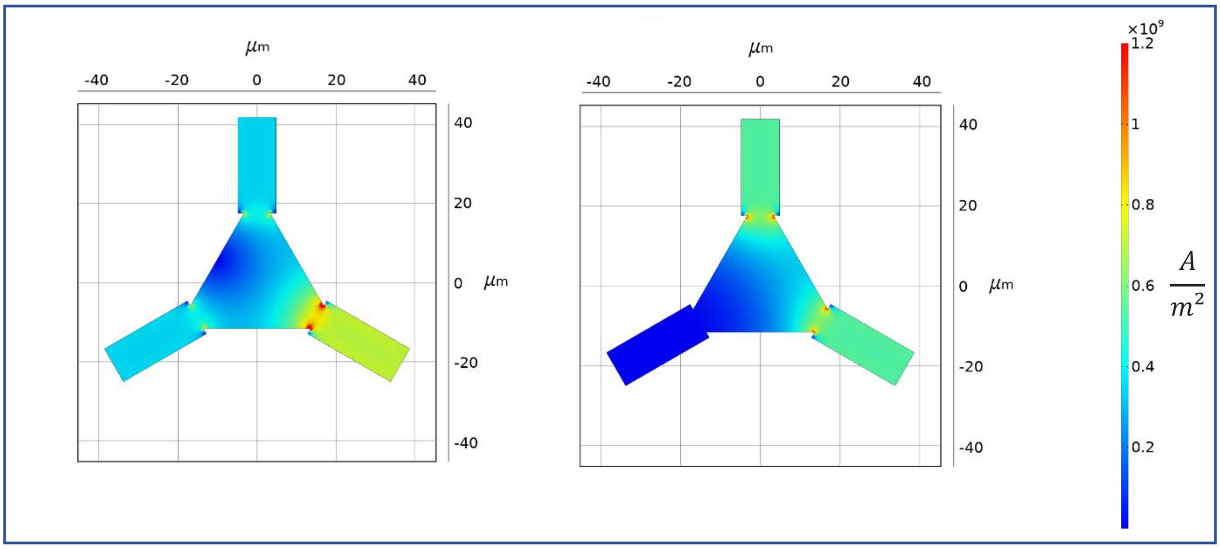

Supplementary Figure 2| Minimalistic COMSOL Multiphysics® simulation of the current density distribution in our device. Left: the current density distribution for one positive potential applied (right bottom) and two grounds. Right: current density distribution for one positive potential (right bottom) one ground (top) and a floating connection (left bottom). The points of highest current density are the (red) bends where the gold contact ends on the stack. Increased current leads to higher temperature here which can result in the annihilation of skyrmion at these points.

Supplementary Figure 3| Multiple skyrmions

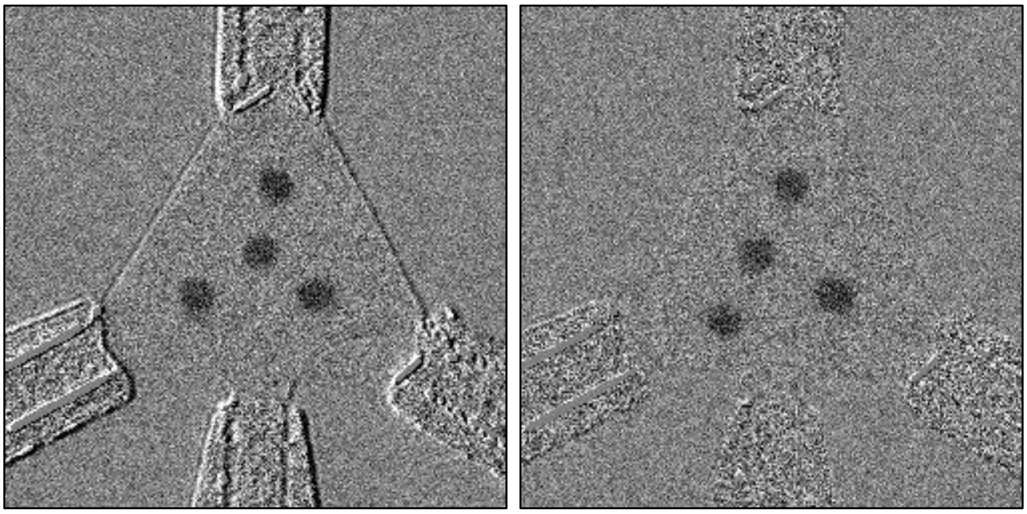

Supplementary Figure 3| Multiple skyrmion states in the device. By using multiple skyrmions the amount of states and thus the capacity of the device increases.

Supplementary Figure 4| Overlap-weighted regional occupation probabilities

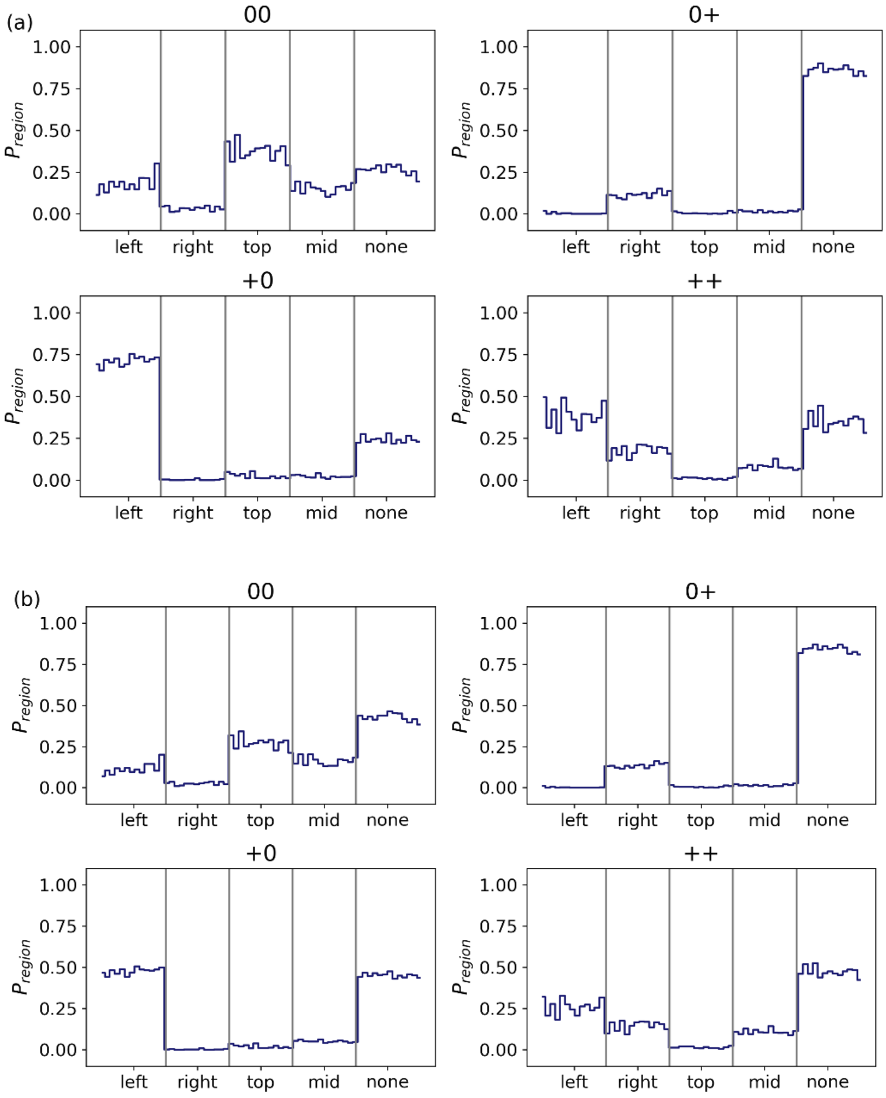

Supplementary Figure 4| Occurrence probabilities in the four read-out regions for all input combinations employed for Boolean logic calculated based on (a) the skyrmion center positions and (b) the relative areal overlap of the skyrmion and the read-out regions. Each bin in each region corresponds to one of the 13 sets of local skyrmion occurrence probabilities.

Supplementary Figure 5| Overlap-weighted logic gates

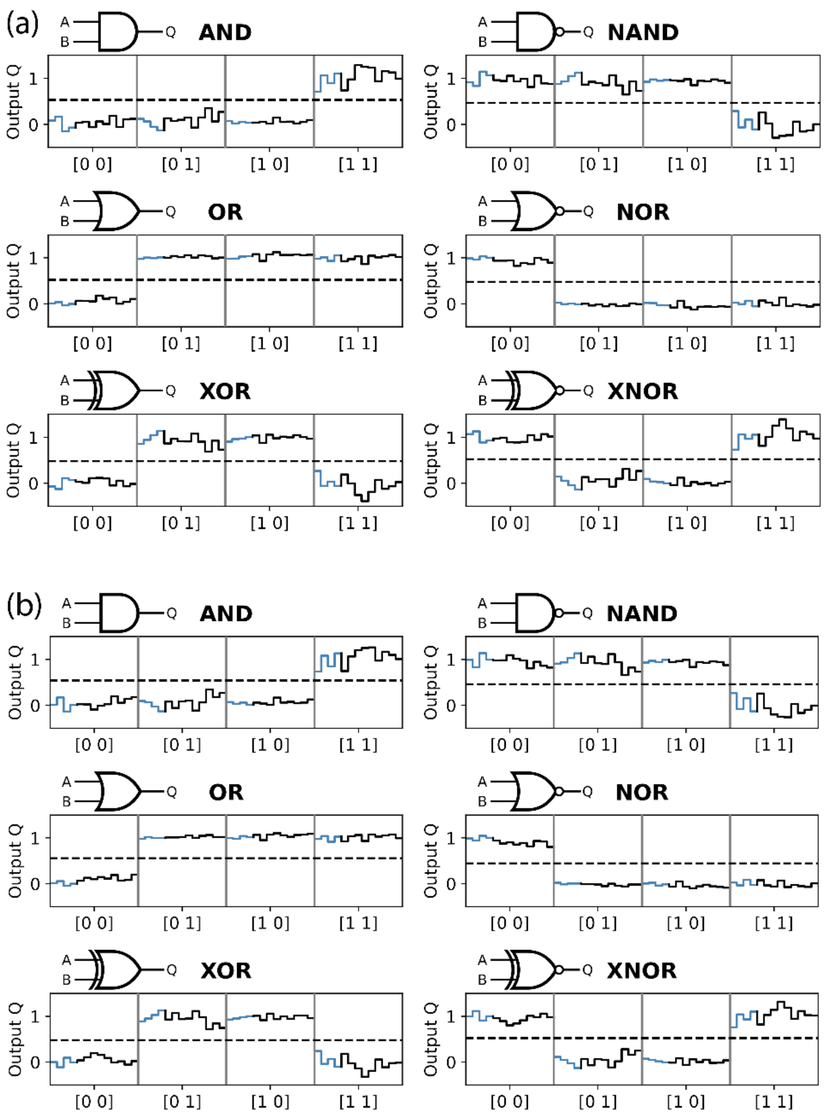

Supplementary Figure 5| Outputs of the linear read-out optimized for different Boolean operations based on local occurrence probabilities calculated using (a) the skyrmion center positions (as in Fig. 3) and (b) the relative areal overlap of the skyrmion and the read-out regions. For each input combination ([0 0], [0 1], [1 0], [1 1]) the output Q of the linear read-out is shown for 13 sets of local skyrmion occurrence probabilities. The light blue and black parts of the curves indicate the sets used for training and testing, respectively. The dashed horizontal line indicates a possible threshold for perceptron read-out.

Supplementary Figure 6| Logic gates with halved time interval for local skyrmion occurrence probabilitiesc

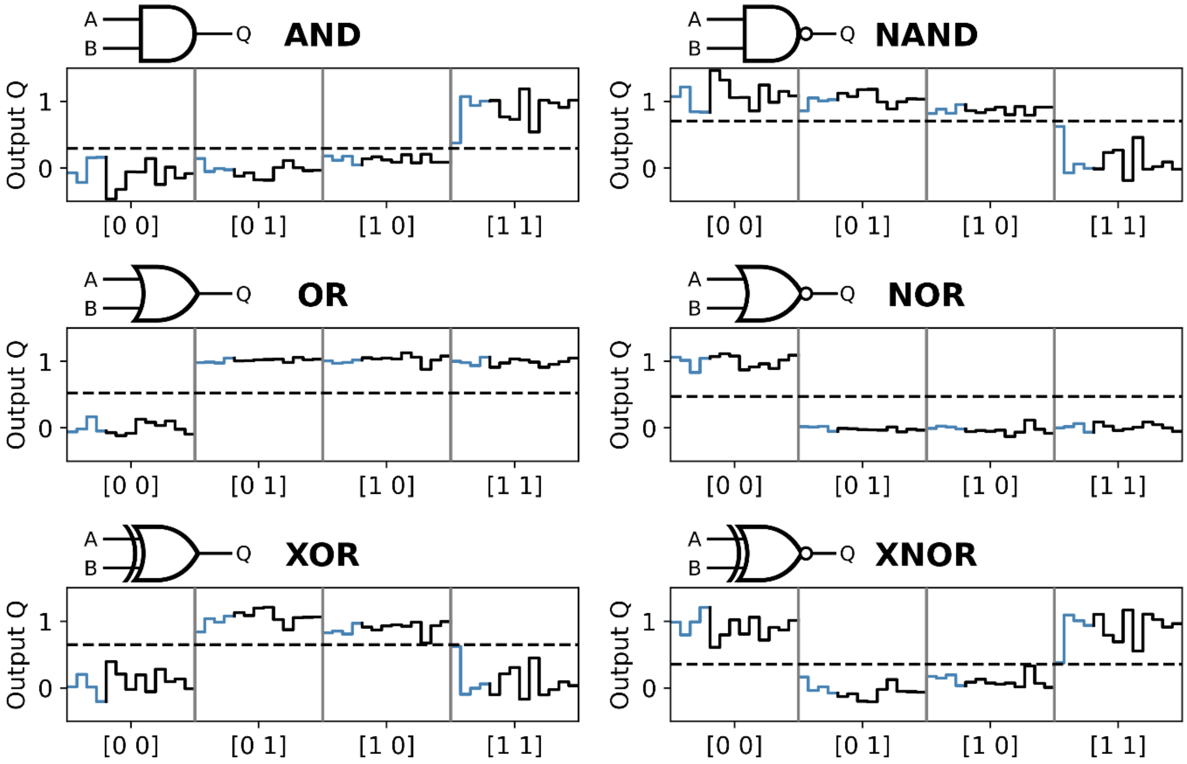

Supplementary Figure 6| Outputs of the linear read-out optimized for different Boolean operations. For each input combination ([0 0], [0 1], [1 0], [1 1]) the output Q of the linear read-out is shown for 13 sets of local skyrmion occurrence probabilities. Compared to Fig. 3, the time interval used to obtain the local skyrmion occurrence probabilities is halved and therefore the SNR is reduced. The light blue and black parts of the curves indicate the sets used for training and testing, respectively. The dashed horizontal line indicates a possible threshold for perceptron read-out. Note that compared to Fig. 3, the minimal distance of the signal from this line is lower.

Supplementary Figure 7| 3-input operations

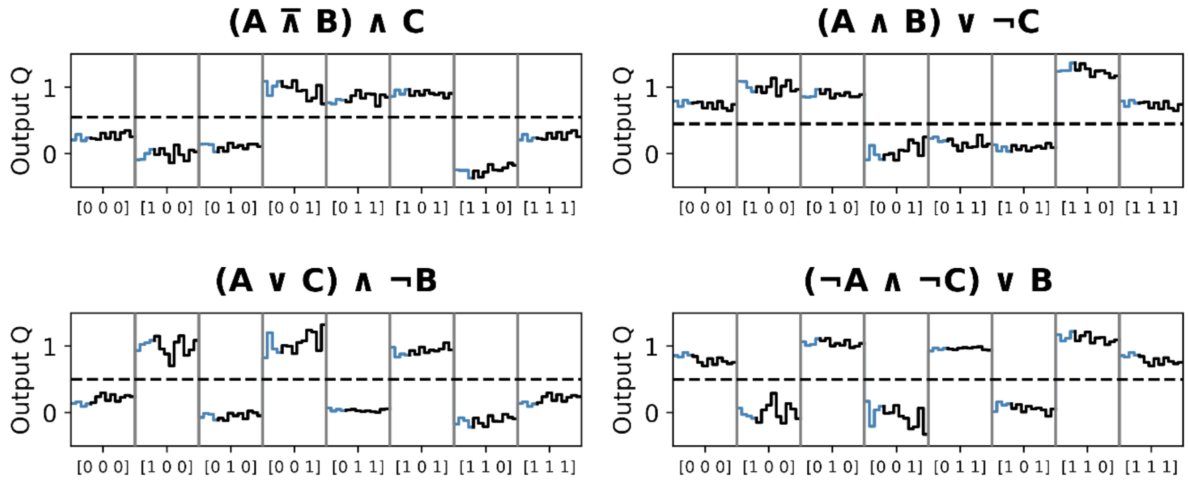

Supplementary Figure 7| Outputs of the linear read-out optimized for different 3-input operations. For each input combination [A B C] the output Q of the linear read-out is shown for 13 sets of local skyrmion occurrence probabilities. The light blue and black parts of the curves indicate the sets used for training and testing, respectively. The dashed horizontal line indicates a possible threshold for perceptron read-out.

Supplementary References

1. Litzius, K. *et al.* Skyrmion Hall effect revealed by direct time-resolved X-ray microscopy. *Nat. Phys.* 13, 170–175 (2017).
2. Miltat, J. *et al.*, Brownian Motion of Magnetic Domain Walls and Skyrmions, and their Diffusion Constants, *Phys. Rev. B* 97 (2018).
3. Zázvorka, J. *et al.* Thermal skyrmion diffusion used in a reshuffler device. *Nat. Nanotechnol.* 14, 658–661 (2019).
4. Liu, H. *et al.*, Ultrafast switching in magnetic tunnel junction based orthogonal spin transfer devices. *Appl. Phys. Lett.* **97**, 242510 (2010).
5. Wu, Y.C. *et al.*, Study of precessional switching speed control in voltage-controlled perpendicular magnetic tunnel junction. *AIP Advances* 10, 035123 (2020).
6. Romera, M. *et al.* Vowel recognition with four coupled spin-torque nano-oscillators. *Nature* 563, 230–234 (2018).
7. COMSOL Multiphysics® v. 5.5. www.comsol.com. COMSOL AB, Stockholm, Sweden.
